# Supplementary material for: Ebf3+ niche-derived CXCL12 is required for the localization and maintenance of hematopoietic stem cells
Source: Nat Commun. 2023 Oct 25;14:6402. doi: 10.1038/s41467-023-42047-2 (PMC10600098; doi:10.1038/s41467-023-42047-2)
Supplement: Supplementary file 1 — Supplementary Information [file 41467_2023_42047_MOESM1_ESM.pdf]

Supplementary Information.

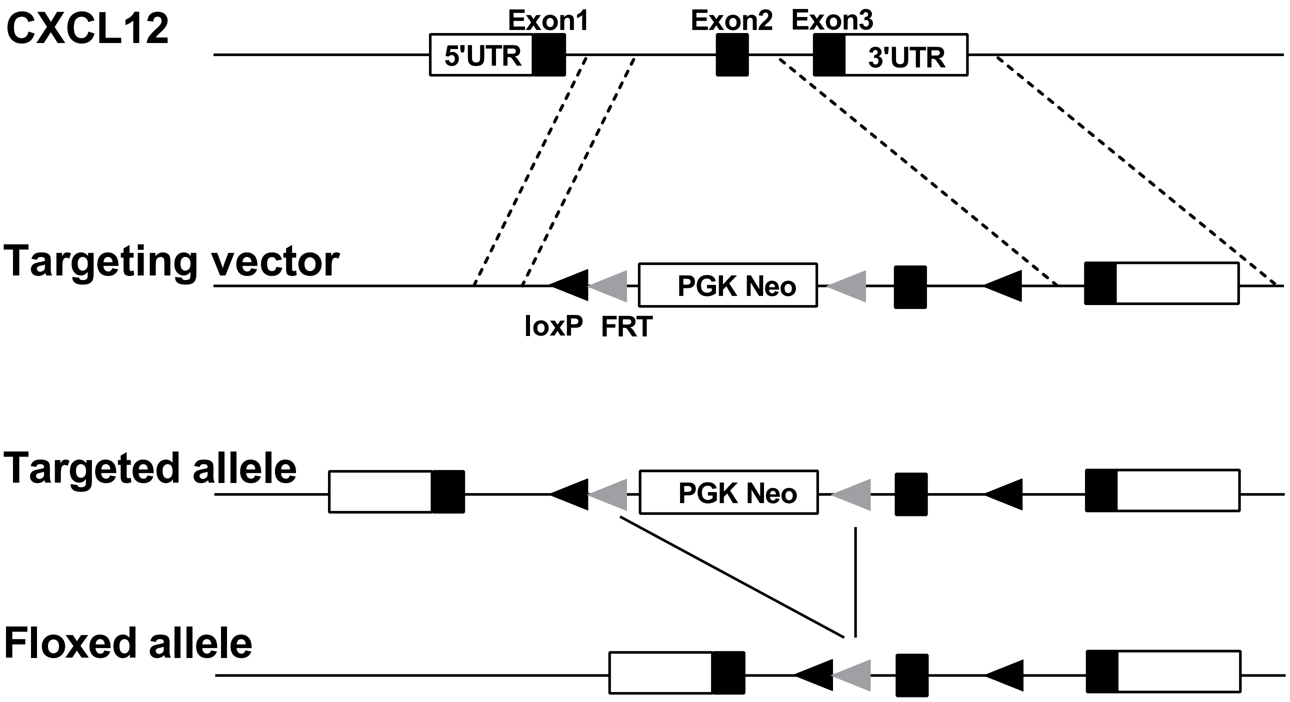

Supplementary Fig. 1 Construction of CXCL12$^{f/f}$ mice.

Targeting strategy to generate a CXCL12 floxed mouse line (CXCL12$^{f/f}$). UTR, untranslated region; PGK, phosphoglycerate kinase.

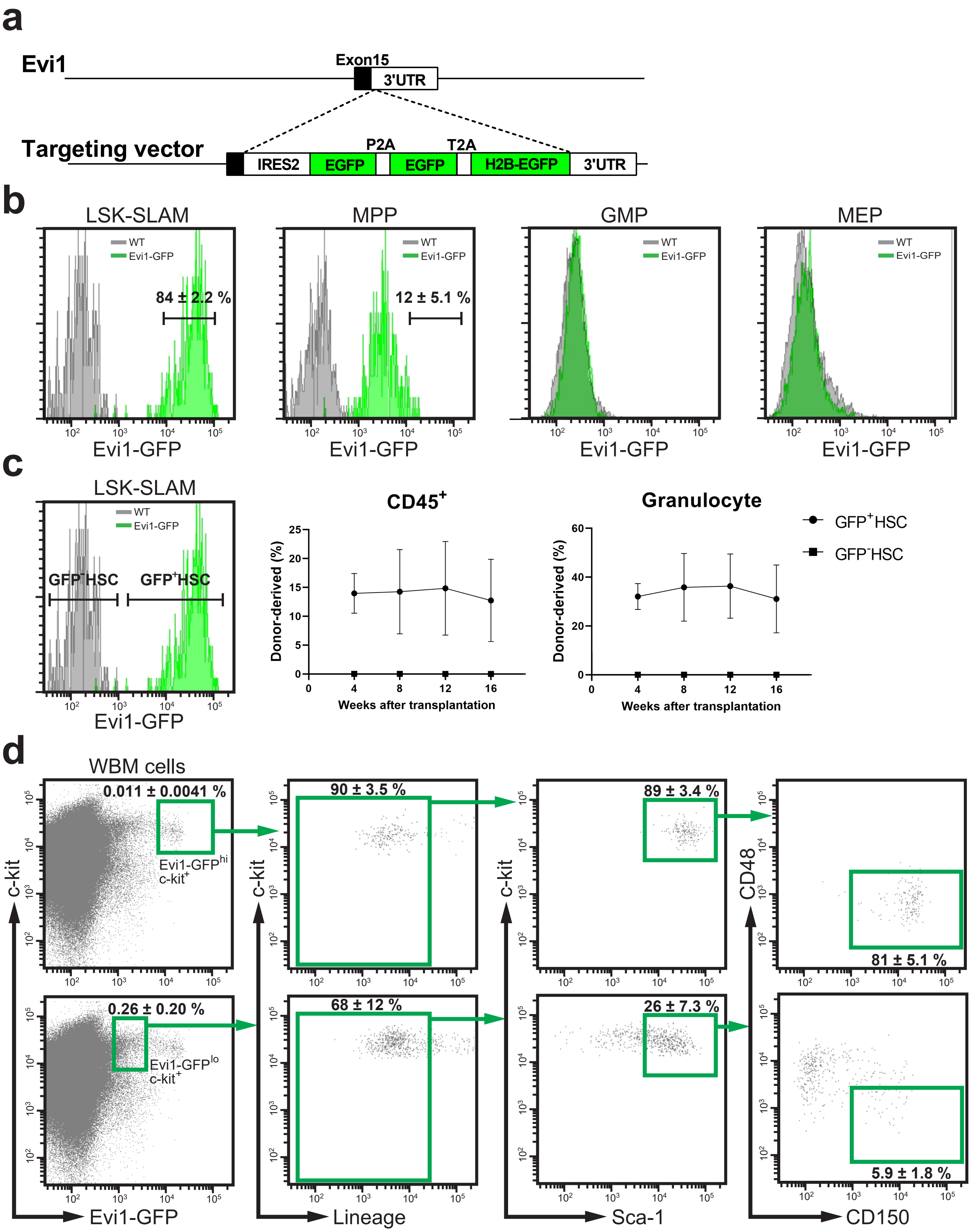

e

Donor cell population	Donor cell dose	Reconstituted mice / Transplanted mice	HSC frequency (95% CI)
Evi1-GFP^{hi} c-kit^{+}	3<br>10<br>50	9/15<br>7/8<br>1/1	1 in 3.8<br>(1/6.7 - 1/2.7)

Supplementary Fig. 2 Evi1-GFP mice allowed visualization of HSCs.

a Targeting strategy to generate a triple-EGFP Evi1 knock-in mouse reporter line (Evi1-GFP).

b Flow cytometric analysis of Evi1-GFP expression in hematopoietic stem and progenitor cells, including LSK-SLAM HSCs, MPPs, GMPs, and MEPs from Evi1-GFP mice.Values represent means ± SD of frequencies of Evi1-GFP^{hi} cells (n = 6).

c Sorted 200 cells in Evi1-GFP^{+}CD150^{+}CD48^{-}LSK or Evi1-GFP^{-}CD150^{+}CD48^{-}LSK populations from 18-week-old CD45.2^{+} Evi1-GFP mice were transplanted along with 5×10^{5} CD45.1^{+} competitor bone marrow cells into CD45.1^{+}CD45.2^{+} wild-type mice. Flow cytometric analysis of Evi1-GFP expression in LSK-SLAM HSCs from wild-type mice and Evi1-GFP mice (left). Donor chimerism of CD45^{+} cells (middle) and granulocytes (right) in the peripheral blood (n = 7). All error bars represent SD of the mean.

d Frequencies of LSK-SLAM HSCs in Evi1-GFP^{hi}c-kit^{+} cells (top panel) and Evi1-GFP^{lo}c-kit^{+} cells (bottom panel). Values represent means ± SD (n = 6).

e Limiting dilution analysis of Evi1-GFP^{hi}c-kit^{+} cells mixed with 5×10^{5} bone marrow cells. The data reflect means from 2 independent experiments. CI, confidence interval.

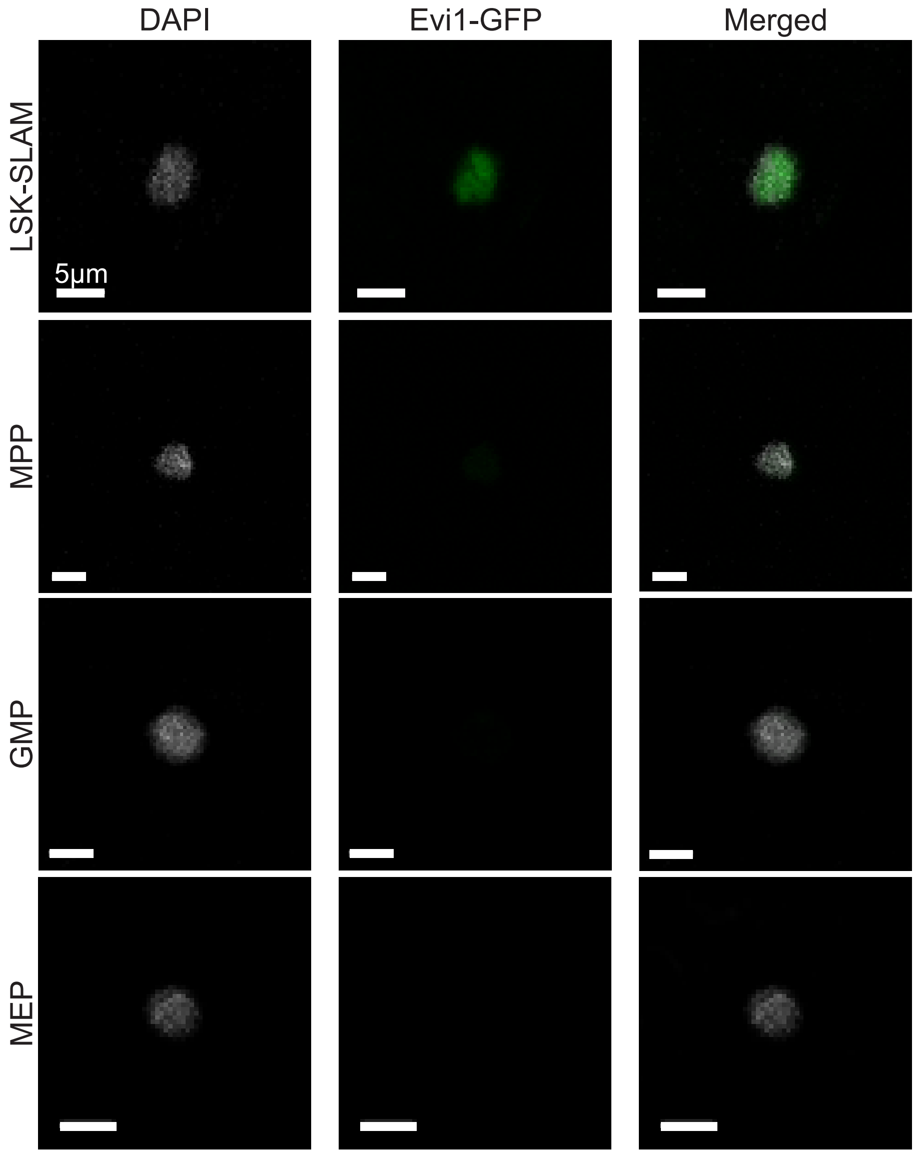

Supplementary Fig. 3 Evi1-GFP fluorescence signal was detected specifically in HSCs by histology.

Confocal microscopy images showing fluorescence of Evi1-GFP in fixed LSK-SLAM HSCs, MPPs, GMPs, and MEPs.

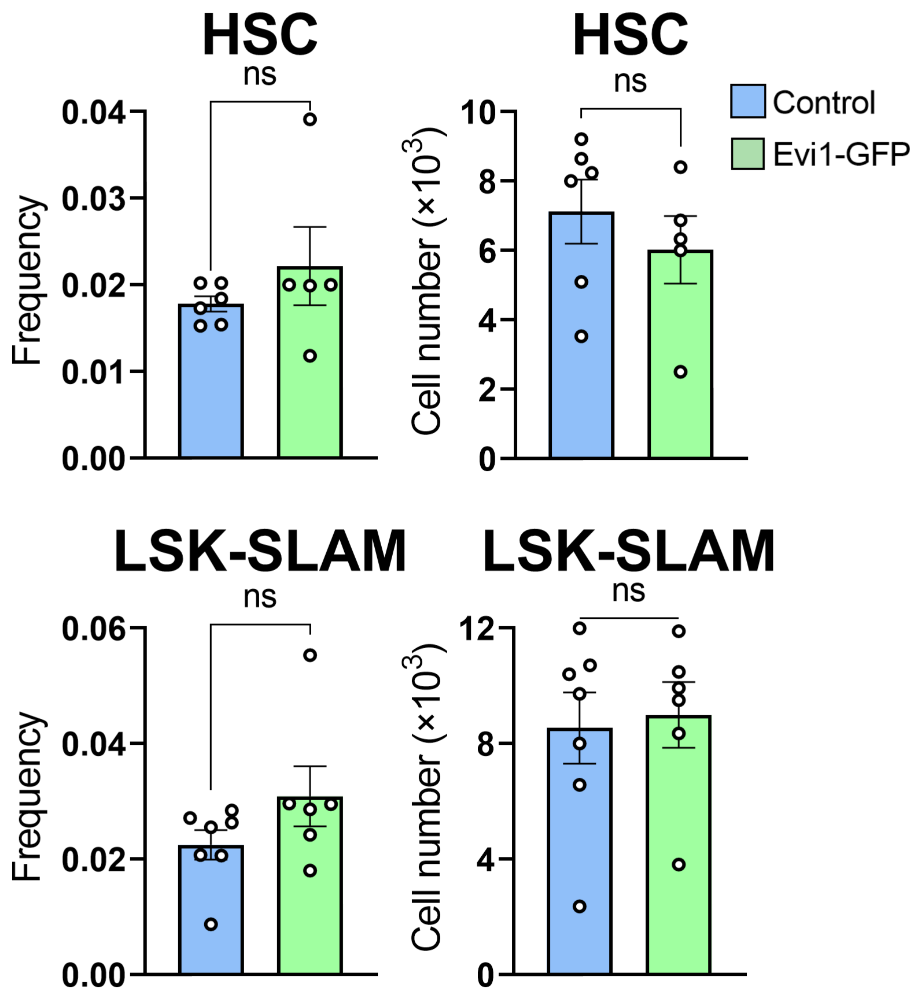

Supplementary Fig. 4 HSC numbers were unaltered in Evi1-GFP mice.

Frequencies and numbers of $CD34^-CD150^+CD48^-$LSK HSCs in the bone marrow of wild-type control (n = 6) and Evi1-GFP (n = 5) mice and those of LSK-SLAM HSCs in the bone marrow of wild-type control (n = 7) and Evi1-GFP (n = 6) mice are shown. All error bars represent SE of the mean. Statistical significances were calculated using the two-tailed unpaired Student’ s t-test. Source data are provided as a Source Data file.

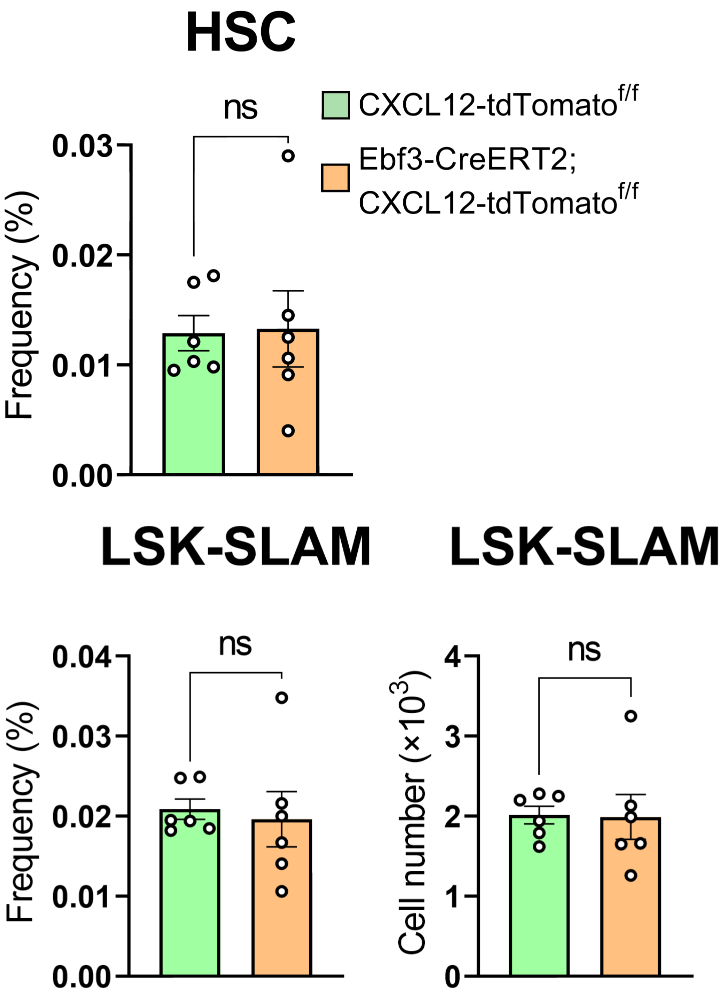

Supplementary Fig. 5 Frequencies of phenotypic LT-HSCs was unaltered in Ebf3-CreERT2;CXCL12-tdTomato$^{f/f}$ mice transplanted with Evi1-GFP bone marrow cells. Frequencies of phenotypic LT-HSCs in bone marrow of CXCL12-tdTomato$^{f/f}$ control and Ebf3-CreERT2;CXCL12-tdTomato$^{f/f}$ conditional knockout mice injected once with tamoxifen are shown (n = 6). All error bars represent SE of the mean. Statistical significances were calculated using the two-tailed unpaired Student' s t-test. Source data are provided as a Source Data file.

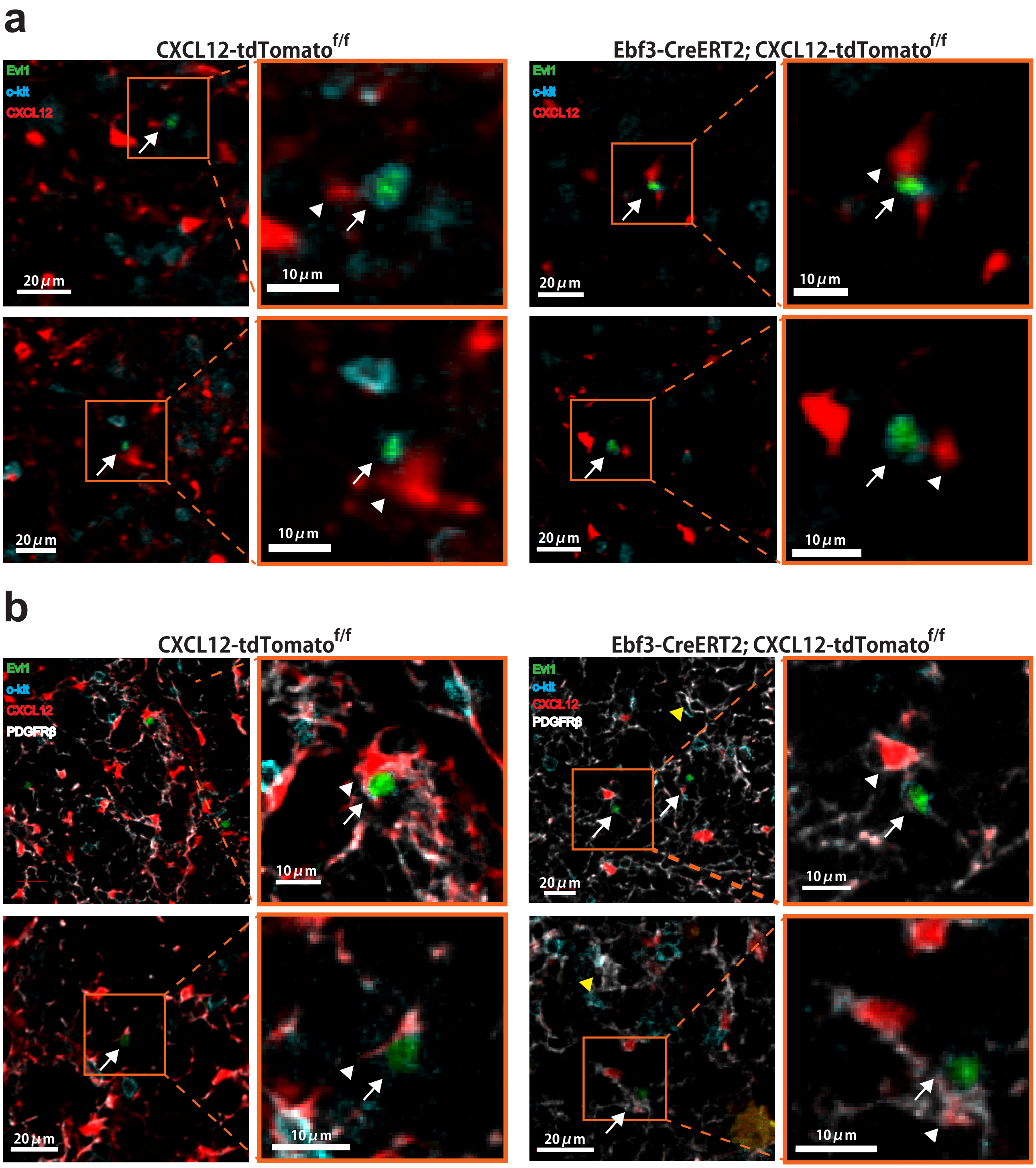

Supplementary Fig. 6 Association of HSCs with CXCL12-deficient CAR cells or CXCL12-intact CAR cells.

a Immunohistochemical analysis of Evi1-GFP^{hi}c-kit^{+} HSCs (arrow) in contact with CXCL12-intact CAR cells (arrowhead).

b Immunohistochemical analysis of Evi1-GFP^{hi}c-kit^{+} HSCs (white arrows) in contact with the CXCL12-tdTomato^{+}PDGFRβ^{+} CXCL12-intact CAR cells (white arrowheads) and distant from the CXCL12-tdTomato^{-}PDGFRβ^{+} CXCL12-deficient CAR cells (yellow arrowheads) in control CXCL12-tdTomato$^{f/f}$ and Ebf3-CreERT2;CXCL12-tdTomato$^{f/f}$ mice.

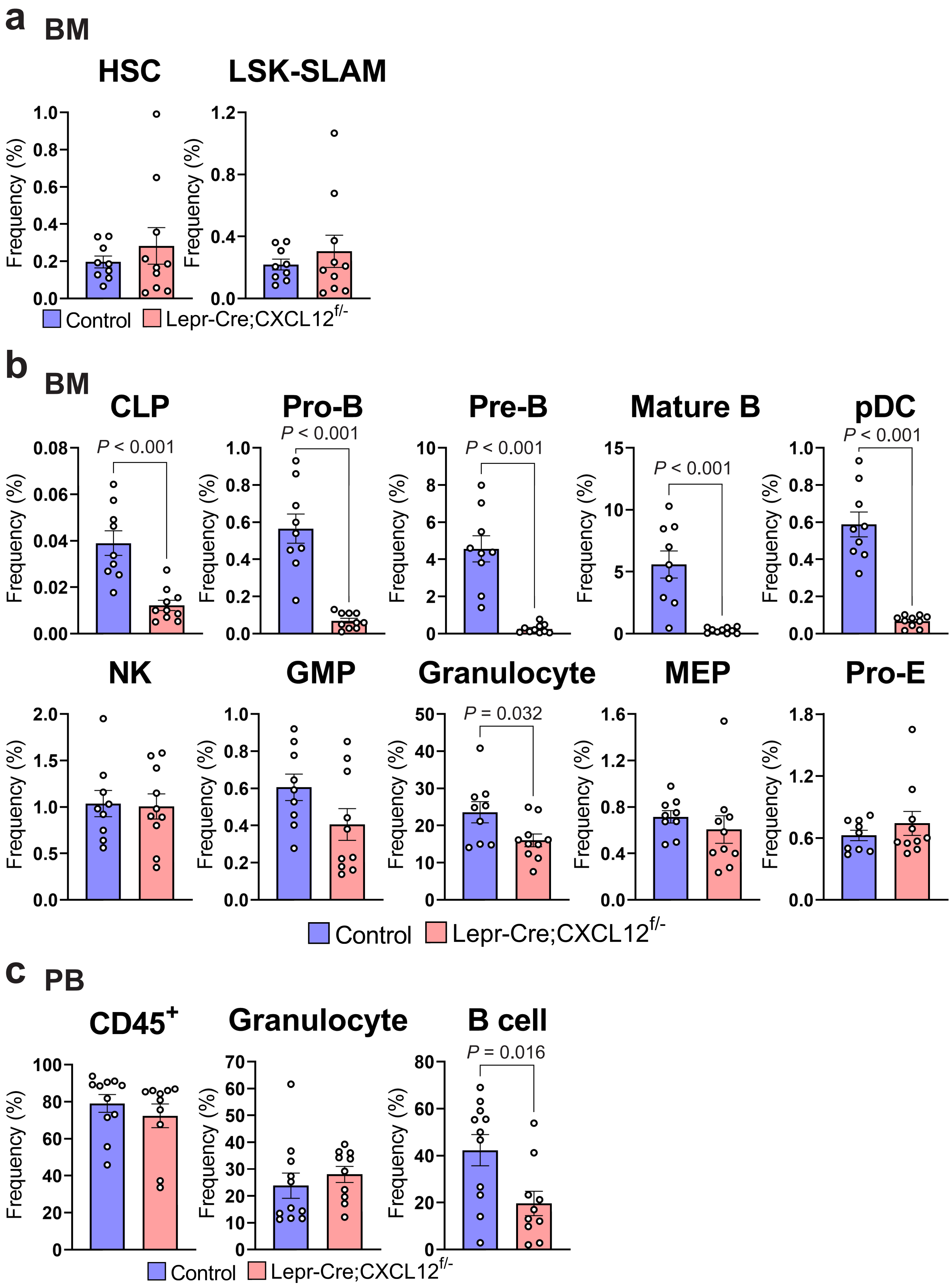
a
BM
HSC
LSK-SLAM
Frequency (%)
Control
Lepr-Cre;CXCL12f/-
b
BM
CLP
Pro-B
Pre-B
Mature B
pDC
P < 0.001
P = 0.032
NK
GMP
Granulocyte
MEP
Pro-E
c
PB
CD45+
Granulocyte
B cell
P = 0.016

Supplementary Fig. 7 Frequencies of hematopoietic cell populations in aged LepR-Cre;CXCL12$^{f/-}$ mice.

a-**c** Bone marrow from 90-week-old LepR-Cre;CXCL12$^{+/+}$ control (n = 9) and LepR-Cre;CXCL12$^{f/-}$ conditional knockout (n = 10) mice and peripheral blood from 90-week-old LepR-Cre;CXCL12$^{+/+}$ control (n = 11) and LepR-Cre;CXCL12$^{f/-}$ conditional knockout (n = 10) were analyzed. Frequencies CD34^{-}CD150^{+}CD48^{-}LSK HSCs, LSK-SLAM HSCs (**a**), CLPs, pro-B cells, pre-B cells, mature B cells, pDCs, NK cells, GMPs, granulocytes, MEPs, and proerythroblasts (pro-E) (**b**) in the bone marrow. Frequencies of CD45^{+} cells, granulocytes, and B cells in the peripheral blood (**c**).

All error bars represent SE of the mean. Statistical significances were calculated using the two-tailed unpaired Student' s t-test. Source data are provided as a Source Data file.

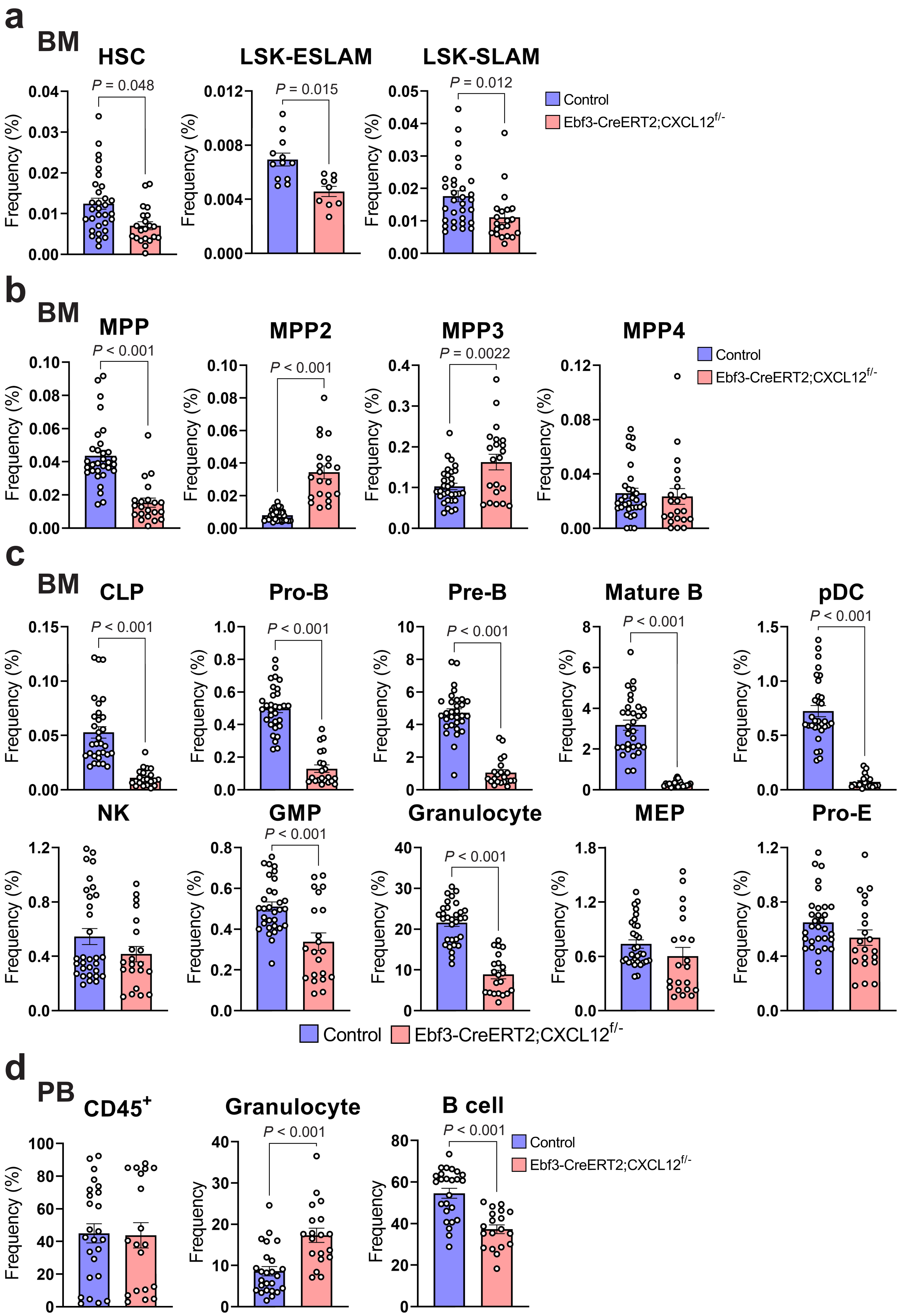
a
BM
HSC
P = 0.048
LSK-ESLAM
P = 0.015
LSK-SLAM
P = 0.012
Control
Ebf3-CreERT2;CXCL12f/-
Frequency (%)
b
BM
MPP
P < 0.001
MPP2
P < 0.001
MPP3
P = 0.0022
MPP4
Control
Ebf3-CreERT2;CXCL12f/-
c
BM
CLP
P < 0.001
Pro-B
P < 0.001
Pre-B
P < 0.001
Mature B
P < 0.001
pDC
P < 0.001
NK
GMP
P < 0.001
Granulocyte
P < 0.001
MEP
Pro-E
Control
Ebf3-CreERT2;CXCL12f/-
d
PB
CD45+
Granulocyte
P < 0.001
B cell
P < 0.001
Control
Ebf3-CreERT2;CXCL12f/-
Frequency

Supplementary Fig. 8 Frequencies of hematopoietic cell populations in mice lacking CXCL12 in CAR cells.

a-**d** Bone marrow from 21- to 25-week-old Ebf3-CreERT2;CXCL12$^{+/+}$ control (n = 31) or CXCL12$^{\Delta CAR}$ (n = 21) mice and peripheral blood from 21- to 25-week-old Ebf3-CreERT2;CXCL12$^{+/+}$ control (n = 25) or CXCL12$^{\Delta CAR}$ (n = 19) mice injected with tamoxifen eight times were analyzed. Frequencies of CD34^{-}CD150^{+}CD48^{-}LSK HSCs, LSK-ESLAM HSCs, LSK-SLAM HSCs (**a**), MPPs, MPP2s, MPP3s, MPP4s (**b**), CLPs, pro-B cells, pre-B cells, mature B cells, pDCs, NK cells, GMPs, granulocytes, MEPs and proerythroblasts (pro-E) (**c**) in the bone marrow. Frequencies of CD45^{+} cells, granulocytes, and B cells in the peripheral blood (**d**).

All error bars represent SE of the mean. Statistical significances were calculated using the two-tailed unpaired Student’ s t-test. Source data are provided as a Source Data file.

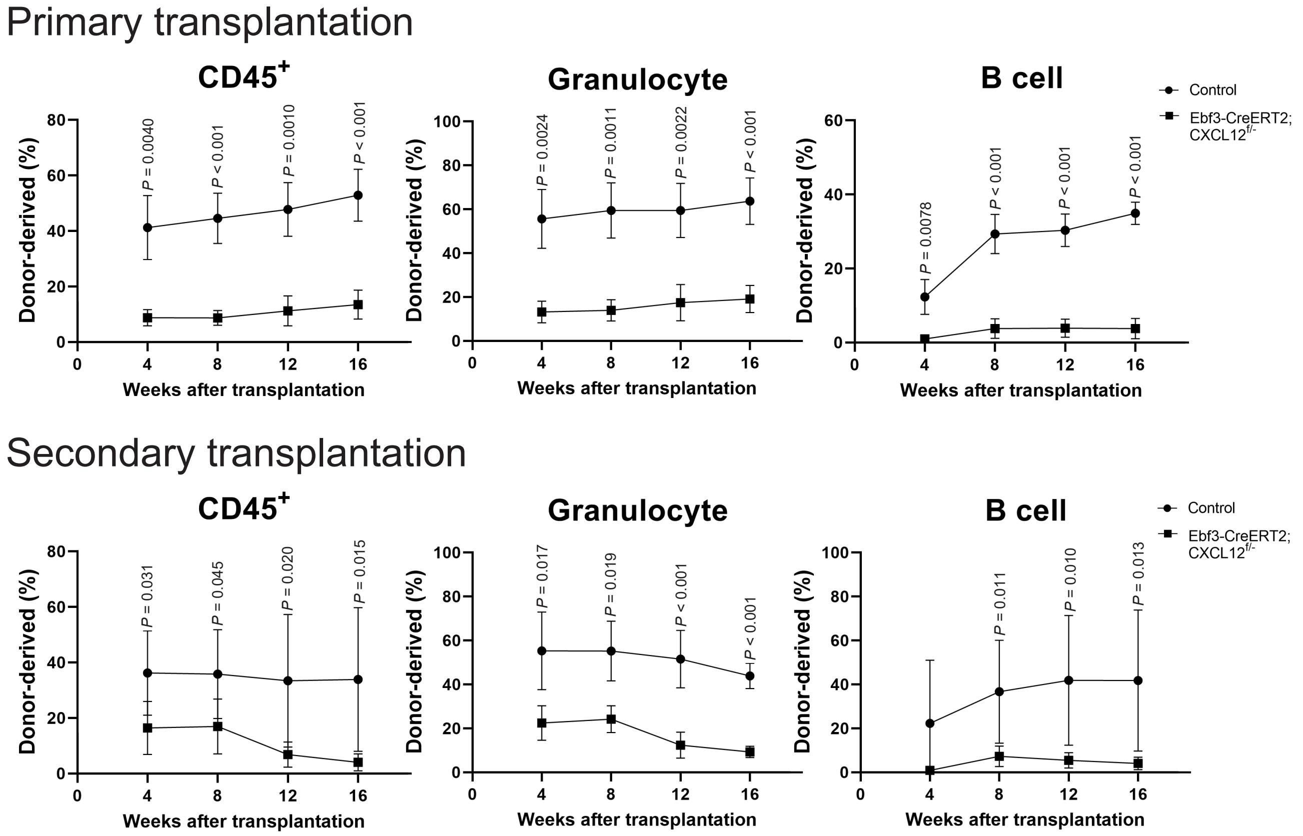

Supplementary Fig. 9 The reconstitution ability of HSCs in mice lacking CXCL12 in CAR cells in primary and secondary recipients.

Sorted 200 cells in the HSC population from Ebf3-CreERT2;CXCL12$^{+/+}$ control or Ebf3-CreERT2;CXCL12$^{f/-}$ CXCL12$^{\Delta CAR}$ mice were transplanted with 5×10^{5} CD45.1^{+} competitor bone marrow cells into CD45.1^{+}CD45.2^{+} wild-type mice. 1500 donor-derived Lin^{-}Sca-1^{+}c-kit^{+} primitive hematopoietic stem and progenitor cells (HSPCs) from primary recipients were transplanted with 1×10^{6} competitor bone marrow cells into secondary recipients. Donor chimerism of CD45^{+} cells, granulocytes, and B cells in the peripheral blood of primary recipients transplanted with HSCs from Ebf3-CreERT2;CXCL12$^{+/+}$ control (n = 6) or CXCL12$^{\Delta CAR}$ (n = 3) mice and secondary recipients transplanted with HSPCs from primary recipients transplanted with HSCs from Ebf3-CreERT2;CXCL12$^{+/+}$ control (n = 4) or CXCL12$^{\Delta CAR}$ (n = 8) mice at 4, 8, 12, 16 weeks after transplantation are shown. All error bars represent SD of the mean. Statistical significances were calculated using the two-tailed unpaired Student' s t-test. Source data are provided as a Source Data file.

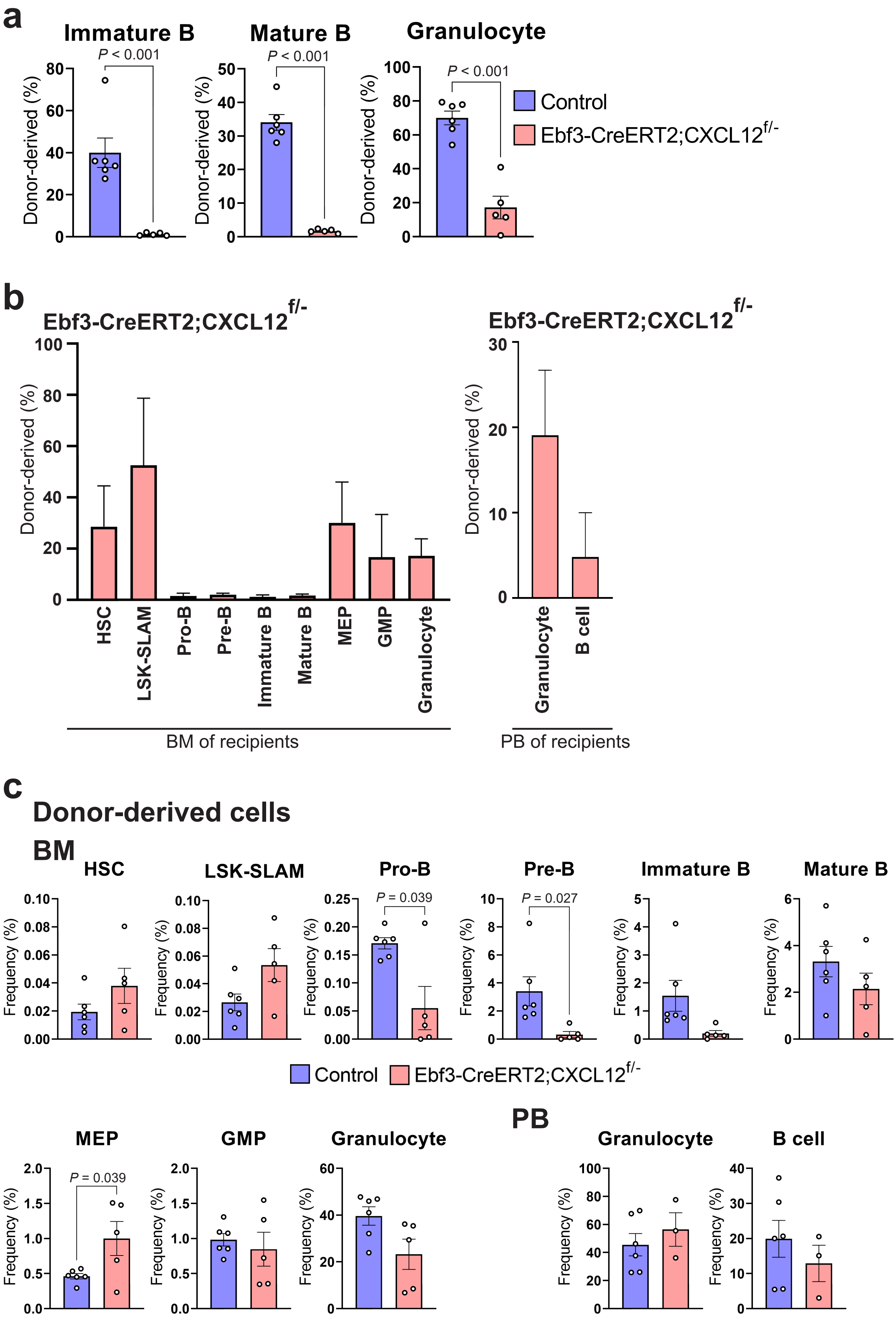
a
Immature B
Mature B
Granulocyte
P < 0.001
Donor-derived (%)
Control
Ebf3-CreERT2;CXCL12f/-
b
Ebf3-CreERT2;CXCL12f/-
Donor-derived (%)
HSC
LSK-SLAM
Pro-B
Pre-B
Immature B
Mature B
MEP
GMP
Granulocyte
B cell
BM of recipients
PB of recipients
c
Donor-derived cells
BM
PB
Frequency (%)
P = 0.039
P = 0.027
P = 0.039

Supplementary Fig. 10 The ability of HSCs to generate B cell progenitors in mice lacking CXCL12 in CAR cells.

200 cells in the HSC population from Ebf3-CreERT2;CXCL12$^{+/+}$ control or Ebf3-CreERT2;CXCL12$^{f/-}$ CXCL12$^{\Delta CAR}$ mice were transplanted with 5×10^{5} CD45.1^{+} competitor bone marrow cells into CD45.1^{+}CD45.2^{+} wild-type mice.

a Donor chimerism of immature B cells, mature B cells, and granulocytes in the bone marrow of recipients transplanted with HSCs from Ebf3-CreERT2;CXCL12$^{+/+}$ control (n = 6) or CXCL12$^{\Delta CAR}$ (n = 5) mice. All error bars represent SE of the mean. Statistical significances were calculated using the two-tailed unpaired Student' s t-test. Source data are provided as a Source Data file.

b Summary of frequencies of donor-derived cells in hematopoietic cell populations in the bone marrow (n = 5) and peripheral blood (n = 3) of recipients transplanted with HSCs from CXCL12$^{\Delta CAR}$ mice. All error bars represent SD of the mean.

c Frequencies of hematopoietic cell populations within donor-derived cells in the bone marrow of recipients transplanted with HSCs from Ebf3-CreERT2;CXCL12$^{+/+}$ control (n = 6) or CXCL12$^{\Delta CAR}$ (n = 5) mice and in the peripheral blood of recipients transplanted with HSCs from Ebf3-CreERT2;CXCL12$^{+/+}$ control (n = 6) or CXCL12$^{\Delta CAR}$ (n = 3) mice. All error bars represent SE of the mean. Statistical significances were calculated using the two-tailed unpaired Student' s t-test. Source data are provided as a Source Data file.

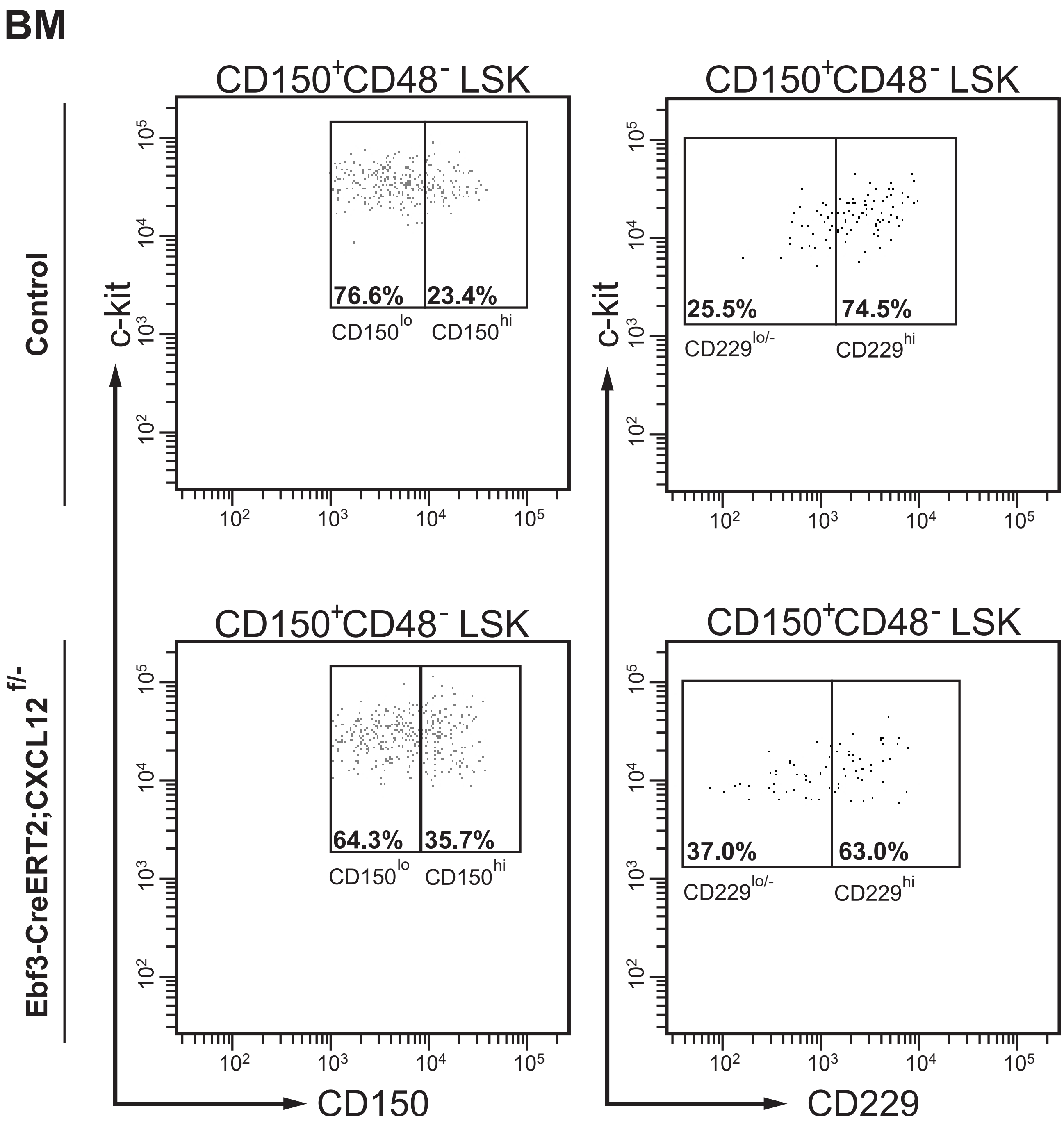

Supplementary Fig. 11 Flow cytometry gating strategies for CD150^{lo} and CD229^{hi} subsets of LSK-SLAM HSCs.

Flow cytometry gating strategies for CD150^{lo} and CD229^{hi} subsets of LSK-SLAM HSCs in the bone marrow of Ebf3-CreERT2;CXCL12$^{+/+}$ control and Ebf3-CreERT2;CXCL12$^{f/-}$ CXCL12$^{\Delta CAR}$ mice.

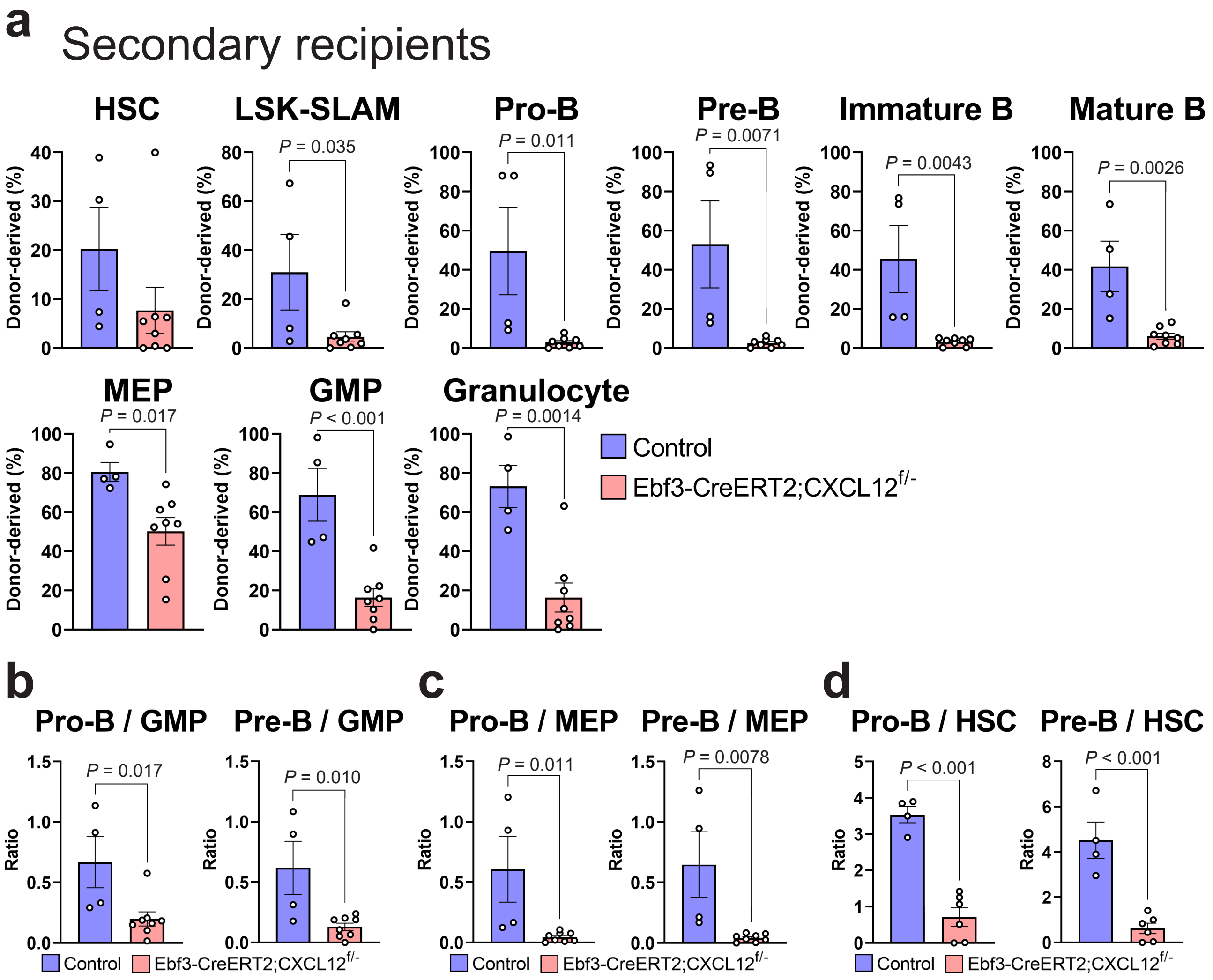

Supplementary Fig. 12 The ability of HSCs from mice lacking CXCL12 in CAR cells to generate B cell progenitors after secondary transplantation.

1500 donor-derived $Lin^-Sca\text{-}1^+c\text{-}kit^+$ primitive hematopoietic stem and progenitor cells from primary recipients transplanted with 200 cells in the HSC population from Ebf3-CreERT2;$CXCL12^{+/+}$ control (n = 4) or $CXCL12^{\Delta CAR}$ (n = 8) mice were transplanted with 1×10^6 competitor bone marrow cells into secondary recipients.

a Donor chimerism of hematopoietic cell populations in the bone marrow of secondary recipients.

b-**d** Donor pro-B/GMP and pre-B/GMP reconstitution ratios (**b**), donor pro-B/MEP and pre-B/MEP reconstitution ratios (**c**), and donor pro-B/HSC and pre-B/HSC reconstitution ratios (**d**) in the bone marrow of secondary recipients.

All error bars represent SE of the mean. Statistical significances were calculated using the two-tailed unpaired Student' s t-test. Source data are provided as a Source Data file.

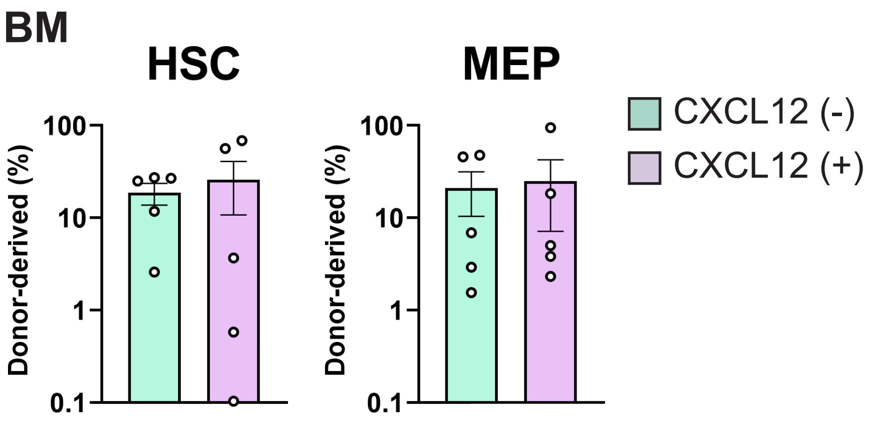

Supplementary Fig. 13 Frequencies of donor cell contribution within hematopoietic cell populations in recipients after transplantation of cultured HSCs.

Sorted 50 cells in the HSC population from CD45.2$^+$ wild-type mice were cultured for 28 days in the presence of SCF and TPO with or without CXCL12, and 75% of cells in each well were transplanted with 1×10^6 CD45.1$^+$ competitor bone marrow cells into CD45.1$^+$CD45.2$^+$ wild-type mice. Donor chimerism of CD34$^-$CD150$^+$CD48$^-$LSK HSCs and MEPs in the bone marrow of recipients at 16 weeks after transplantation is shown (n = 5). All error bars represent SE of the mean.

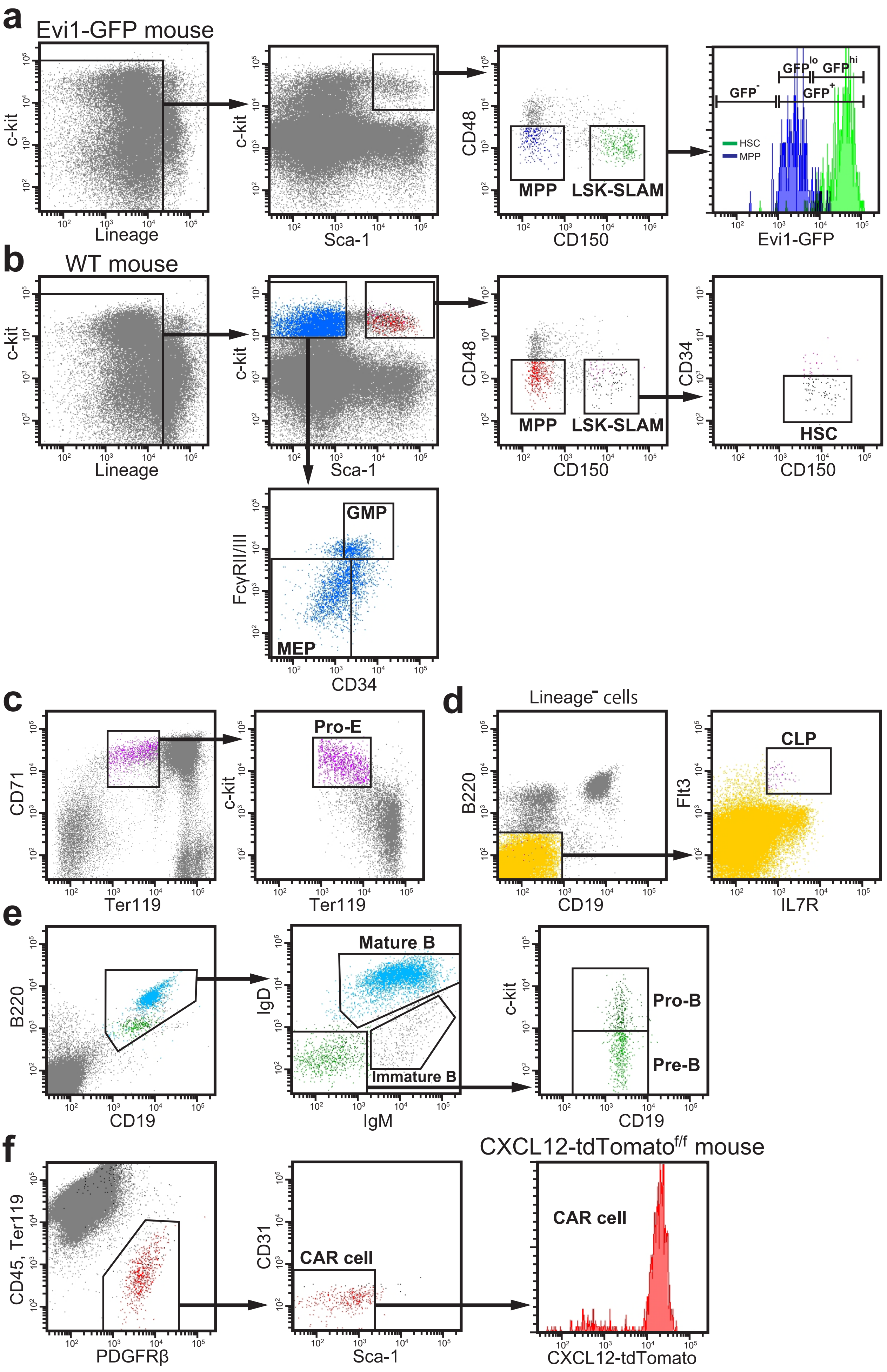
a
Evi1-GFP mouse
c-kit
Lineage
Sca-1
CD48
CD150
MPP
LSK-SLAM
GFP-
GFPlo
GFPhi
GFP+
HSC
MPP
Evi1-GFP
b
WT mouse
CD34
HSC
FcγRII/III
GMP
MEP
c
CD71
Ter119
Pro-E
d
Lineage- cells
B220
CD19
Flt3
IL7R
CLP
e
IgD
IgM
Mature B
Immature B
Pro-B
Pre-B
f
CXCL12-tdTomatof/f mouse
CD45, Ter119
PDGFRβ
CD31
CAR cell
CXCL12-tdTomato

Supplementary Fig. 14 Flow cytometry gating strategies for hematopoietic stem and progenitor cells and CAR cells.

a Flow cytometry gating strategies for LSK-SLAM HSCs and MPPs in an Evi1-GFP mouse.

b-**f** Flow cytometry gating strategies for phenotypic LT-HSCs, MPPs, MEPs, GMPs (**b**), proerythroblasts (pro-E) (**c**), CLPs (**d**), pro-B cells, pre-B cells (**e**), and CAR cells (**f**).

Supplementary Table 1 Antibodies used for flow cytometry.

Antibody	Clone	Conjugated	Company
B220	RA3-6B2	Pacific Blue	Biolegend
B220	RA3-6B2	PE	Biolegend
B220	RA3-6B2	PE-Cy5	eBioscience
B220	RA3-6B2	purified	Biolegend
CD3	145-2C11	APC	Biolegend
CD3	145-2C11	PE-Cy5	Biolegend
CD3	145-2C11	purified	Biolegend
CD4	GK1.5	purified	Biolegend
CD8	53-6.7	purified	Biolegend
CD11b	M1/70	biotin	BD Pharmingen
CD11b	M1/70	FITC	Biolegend
CD11b	M1/70	PE-Cy5	Biolegend
CD11b	M1/70	PE-Cy7	Biolegend
CD11b	M1/70	purified	Biolegend
CD19	1D3	PE	eBioscience
CD31	MEC13.3	Alexa Fluor 647	Biolegend
CD31	MEC13.3	FITC	Biolegend
CD34	RAM34	biotin	eBioscience
CD34	RAM34	FITC	eBioscience
CD45	30-F11	Alexa Fluor 647	Biolegend
CD45	30-F11	PE-Cy5	Biolegend
CD45.1	A20	biotin	Biolegend
CD45.1	A20	FITC	Biolegend
CD45.2	104	APC-eFluor780	eBioscience
CD48	HM48-1	Pacific Blue	Biolegend
CD49b	DX5	PE-Cy7	Biolegend
CD71	C2	PE	BD Pharmingen
CD150	TC15-12F12.2	PE	Biolegend
CD201(EPCR)	eBio1560	biotin	eBioscience
CD229	Ly9ab3	biotin	Biolegend
c-kit	2B8	APC	Biolegend
c-kit	2B8	PE-Cy7	Biolegend
FcγRII/III	2.4G2	V450	BD Pharmingen
Flt3	A2F10	biotin	eBioscience
Flt3	A2F10	PE	Biolegend
Gr-1	RB6-8C5	Pacific Blue	Biolegend
Gr-1	RB6-8C5	PE-Cy5	Biolegend
Gr-1	RB6-8C5	purified	Biolegend
IgDb	217-170	biotin	BD Pharmingen
IgDb	11-26c.2a	FITC	Biolegend
IgM	11/41	APC	eBioscience
IL-7Ra	A7R34	Biotin	eBioscience
IL-7Ra	A7R34	PE-Cy7	Biolegend
Ly6D	49-H4	FITC	Biolegend
NK1.1	PK136	PE	BD Pharmingen
PDCA-1	JF05-1C2.4.1	FITC	Miltenyi Biotech
PDGFR-β	BAF1042	biotin	R&D Systems
Sca-1	E13-161.7	PE-Cy7	Biolegend
Streptavidin		BV421	Biolegend
Streptavidin		BV605	Biolegend
Streptavidin		DyLight649	Biolegend
Ter119	Ter119	APC	Biolegend
Ter119	Ter119	PE-Cy5	Biolegend
Ter119	Ter119	purified	Biolegend

Supplementary Table 2 Antibodies used for immunohistochemical analysis.

Antibody	Clone	Conjugated	Company	Part Number
c-kit	goat polyclonal	purified	R&D Systems	AF1356
S100	EP1576Y	purified	Abcam	Ab52462
Goat IgG	donkey polyclonal	Alexa Fluor 647	Jackson ImmunoRes.	705-606-147
PDGFRβ	goat polyclonal	purified	R&D Systems	AF1042
Rabbit IgG	donkey polyclonal	Alexa Fluor 647	Jackson ImmunoRes.	711-605-152

Supplementary Table 3 Primers used for RT-PCR.

Gene name	Sequence (5' – 3')								
Gapdh	TCA	TGA	GCC	CTT	CCA	CAA	TG		
	GGT	GTG	AAC	CAC	GAG	AAA	TAT		
Cxcl12	CCA	GAG	CCA	ACG	TCA	AGC	AT		
	CAT	CCG	TGC	AAC	AAT	CTG	AA		
Evi1	CTG	AGC	CAA	CCT	CCA	TTA	GTT	CTT	C
	CCT	TGT	CAG	ACA	GTG	ACA	ACA	TCA	
p57	CAG	CGG	ACG	ATG	GAA	GAA	CT		
	CTC	CGG	TTC	CTG	CTA	CAT	GAA		
