# Supplementary material for: Ebf3+ niche-derived CXCL12 is required for the localization and maintenance of hematopoietic stem cells
Source: Nat Commun. 2023 Oct 25;14:6402. doi: 10.1038/s41467-023-42047-2 (PMC10600098; doi:10.1038/s41467-023-42047-2)
Supplement: Supplementary file 3 — Reporting Summary [file 41467_2023_42047_MOESM3_ESM.pdf]

nature portfolio

Corresponding author(s): Takashi Nagasawa

Last updated by author(s): Jul 28, 2023

Reporting Summary

Nature Portfolio wishes to improve the reproducibility of the work that we publish. This form provides structure for consistency and transparency in reporting. For further information on Nature Portfolio policies, see our Editorial Policies and the Editorial Policy Checklist.

Statistics

For all statistical analyses, confirm that the following items are present in the figure legend, table legend, main text, or Methods section.

n/a	Confirmed	
☐	☒	The exact sample size (*n*) for each experimental group/condition, given as a discrete number and unit of measurement
☐	☒	A statement on whether measurements were taken from distinct samples or whether the same sample was measured repeatedly
☐	☒	The statistical test(s) used AND whether they are one- or two-sided<br>*Only common tests should be described solely by name; describe more complex techniques in the Methods section.*
☒	☐	A description of all covariates tested
☐	☒	A description of any assumptions or corrections, such as tests of normality and adjustment for multiple comparisons
☐	☒	A full description of the statistical parameters including central tendency (e.g. means) or other basic estimates (e.g. regression coefficient) AND variation (e.g. standard deviation) or associated estimates of uncertainty (e.g. confidence intervals)
☐	☒	For null hypothesis testing, the test statistic (e.g. *F*, *t*, *r*) with confidence intervals, effect sizes, degrees of freedom and *P* value noted<br>*Give P values as exact values whenever suitable.*
☒	☐	For Bayesian analysis, information on the choice of priors and Markov chain Monte Carlo settings
☒	☐	For hierarchical and complex designs, identification of the appropriate level for tests and full reporting of outcomes
☒	☐	Estimates of effect sizes (e.g. Cohen's *d*, Pearson's *r*), indicating how they were calculated

Our web collection on statistics for biologists contains articles on many of the points above.

Software and code

Policy information about availability of computer code

Data collection	FACS Diva 8.0.1, Zeiss ZEN 3.0 SR, IMARIS 9.0.2
Data analysis	FACS Diva 8.0.1, GraphPad PRISM 9.3.1

For manuscripts utilizing custom algorithms or software that are central to the research but not yet described in published literature, software must be made available to editors and reviewers. We strongly encourage code deposition in a community repository (e.g. GitHub). See the Nature Portfolio guidelines for submitting code & software for further information.

Data

Policy information about availability of data

All manuscripts must include a data availability statement. This statement should provide the following information, where applicable:

- Accession codes, unique identifiers, or web links for publicly available datasets
- A description of any restrictions on data availability
- For clinical datasets or third party data, please ensure that the statement adheres to our policy

Source data are provided with this paper.

Human research participants

Policy information about studies involving human research participants and Sex and Gender in Research.

Reporting on sex and gender	This study did not involve human research participants.
Population characteristics	This study did not involve human research participants.
Recruitment	This study did not involve human research participants.
Ethics oversight	This study did not involve human research participants.

Note that full information on the approval of the study protocol must also be provided in the manuscript.

Field-specific reporting

Please select the one below that is the best fit for your research. If you are not sure, read the appropriate sections before making your selection.

☒ Life sciences ☐ Behavioural & social sciences ☐ Ecological, evolutionary & environmental sciences

For a reference copy of the document with all sections, see nature.com/documents/nr-reporting-summary-flat.pdf

Life sciences study design

All studies must disclose on these points even when the disclosure is negative.

Sample size	No statistical method was used to predetermine sample size.<br>Sample sizes were chosen based on extensive experience with similar experiments in our laboratory.
Data exclusions	No data were excluded.
Replication	All experiments were repeated at least three times with sufficient reproducibility.
Randomization	All samples and animals were analysed and allocated randomly.
Blinding	The investigators were not blinded to allocation during experiments. Blinding was not possible as the same investigator performed genotyping and analysis.

Reporting for specific materials, systems and methods

We require information from authors about some types of materials, experimental systems and methods used in many studies. Here, indicate whether each material, system or method listed is relevant to your study. If you are not sure if a list item applies to your research, read the appropriate section before selecting a response.

Materials & experimental systems

n/a	Involved in the study	
☐	☒	Antibodies
☒	☐	Eukaryotic cell lines
☒	☐	Palaeontology and archaeology
☐	☒	Animals and other organisms
☒	☐	Clinical data
☒	☐	Dual use research of concern

Methods

n/a	Involved in the study	
☒	☐	ChIP-seq
☐	☒	Flow cytometry
☒	☐	MRI-based neuroimaging

Antibodies

Antibodies used	B220-PE/Cy5 (1:800, RA3-6B2, 15-0452-83, eBioscience), B220-PE (1:200, RA3-6B2, 103208, Biolegend), B220-PB (1:100, RA3-6B2, 103227, Biolegend), B220 purified (1:200, RA3-6B2, 103202, Biolegend), CD3ε-PE/Cy5 (1:400, 145-2C11, 100310, Biolegend), CD3ε-APC (1:100, 145-2C11, 100312, Biolegend), CD3ε purified (1:200, 145-2C11, 100302, Biolegend), CD4 purified (1:200, GK1.5, 100402, Biolegend), CD8 purified (1:200, 53-6.7, 100702, Biolegend), CD11b-PE/Cy5 (1:1600, M1/70, 101210, Biolegend), CD11b-PE/Cy7 (1:100, M1/70, 101216, Biolegend), CD11b-FITC (1:100, M1/70, 101206, Biolegend), CD11b-biotin (1:100, M1/70, 5533309, BD Pharmingen), CD11b purified (1:400, M1/70, 101202, Biolegend), CD19-PE (1:100, 1D3, 12-0193-83, eBioscience), CD31-Alexa Fluor

647 (1:200, MEC13.3, 102516, Biolegend), CD31-FITC (1:200, MEC13.3, 102506, Biolegend), CD34-biotin (1:25, RAM34, 13-0341-82, eBioscience), CD34-FITC (1:25, RAM34, 11-0341-85, eBioscience), CD45-Alexa Fluor 647 (1:400, 30-F11, 103124, Biolegend), CD45-PE/Cy5 (1:400, 30-F11, 103110, Biolegend), CD45.1-biotin (1:100, A20, 110704, Biolegend), CD45.1-FITC (1:100, A20, 110704, Biolegend), CD45.2-APC eFluor 780 (1:100, 104, 47-0454-82, eBioscience), CD48-PB (1:200, HM48-1, 103406, Biolegend), CD49b-PE/Cy7 (1:40, DX5, 108922, Biolegend), CD71-PE (1:400, C2, 553267, BD Pharmingen), CD150-PE (1:100, TC15-12F12.2, 115904, Biolegend), CD201-biotin (1:100,eBio1560, 13-2012-82, eBioscience), CD229-biotin (1:100, Ly9ab3, 122903, Biolegend), c-Kit-APC (1:200, 2B8, 2078220, eBioscience), c-Kit-PE/Cy7 (1:200, 2B8, 105814, Biolegend), FcγR II/III-PE (1:100, 2.4G2, 553145, BD Pharmingen), Flt3-Biotin (1:100, A2F10, 135308, Biolegend), Flt3-PE (1:100, A2F10, 135306, Biolegend), Gr-1-PB (1:400, RB6-8C5, 108430, Biolegend), Gr-1-PE/Cy5 (1:800, RB6-8C5, 108410, Biolegend), Gr1 purified (1:200, 1A8, 127602, Biolegend), IgD-FITC (1:200, 11-26c.2a, 405704, Biolegend), IgD-biotin (1:200, 217-170, 553509, BD Pharmingen), IgM-APC (1:100, II/41, 2056825, eBioscience), IL-7Rα-PE/Cy7 (1:100, A7R34, 135014, Biolegend), IL-7Rα-biotin (1:100, A7R34, 13-1271-82, eBioscience), Ly6D-FITC (1:100, 49-H4, 138606, Biolegend), NK1.1-PE (1:100, PK136, 553165, BD Pharmingen),PDCA-1-FITC (1:50, JF05-1C2.4.1, 130-102-229, Miltenyi Biotec), PDGFRβ-Biotin (1:200, BAF1042, R&D), Sca-1-PE/Cy7 (1:100, E13-161.7, 108114, Biolegend), Ter119-PE/Cy5 (1:400, Ter119, 116210, Biolegend), Ter119-APC (1:100, Ter119, 116212, Biolegend), Ter119 purified (1:200, Ter119, 116202, Biolegend), BV421-streptavidin (405225, Biolegend), DyLight 649-streptavidin (405224, Biolegend), BV605-streptavidin (405229, Biolegend), c-kit purified (1:100, goat polyclonal, AF1356, R&D Systems), S100 purified (1:100, EP1576Y, Ab52462, Abcam), PDGFRβ purified (1:200, goat polyclonal, AF1042, R&D Systems), Goat IgG-Alexa Fluor 647 (1:300, donkey polyclonal, 705-606-147, Jackson ImmunoResearch) and Rabbit IgG-Alexa Fluor 647 (1:300, donkey polyclonal, 711-605-152, Jackson ImmunoResearch).

Validation

All antibodies were validated by their manufacturers.
Statements for validation of antibodies can be found from these manufacturers' websites.
Biolegend (https://www.biolegend.com/ja-jp)
eBioscience (https://www.thermofisher.com/jp/ja/home/life-science/cell-analysis/flow-cytometry.html)
BD Pharmingen (https://www.bdbiosciences.com/en-us)
Abcam (https://www.abcam.com/)
Jackson ImmunoResearch (https://www.jacksonimmuno.com/)
R&D Systems (https://www.rndsystems.com/)

Animals and other research organisms

Policy information about studies involving animals; ARRIVE guidelines recommended for reporting animal research, and Sex and Gender in Research

Laboratory animals

Male and female mice were used between 22 weeks to 33 weeks of age, depending on the respective experiments. C57BL/6J mice (Ly5.2) were purchased from Japan SLC (Shizuoka, Japan). Ebf3-CreERT2 mice and CXCL12 +/- mice were generated in our laboratory and have been previously described. CXCL12 f/f mice and CXCl12-tdTomato f/f mice were generated with two loxP sites flanking exon 2 and 3 of Cxcl12 by electroporation of a targeting vector into embryonic stem cells. Evi1-GFP knock-in mice were generated by CRISPR/Cas9 mediated gene targeting. All mice were bred and maintained under specific pathogen-free conditions at the animal facilities of Osaka University. These mice were maintained in 12 hour light/dark cycle, and the housing temperature and humidity were 23±1.5°C and 45±15%, respectively. All mouse strains used are reported in Materials and Methods: "Mice"

Wild animals

This study did not involve wild animals.

Reporting on sex

Sex wasn't considered in the study design.

Field-collected samples

This study did not involve field-collected samples.

Ethics oversight

All animal experiments were performed in accordance with approved protocols of the Institutional Animal Care and Use Committees at Osaka University and Kyoto University.

Note that full information on the approval of the study protocol must also be provided in the manuscript.

Flow Cytometry

Plots

Confirm that:

☒ The axis labels state the marker and fluorochrome used (e.g. CD4-FITC).

☒ The axis scales are clearly visible. Include numbers along axes only for bottom left plot of group (a 'group' is an analysis of identical markers).

☒ All plots are contour plots with outliers or pseudocolor plots.

☒ A numerical value for number of cells or percentage (with statistics) is provided.

Methodology

Sample preparation

Bone marrow cells were isolated by flushing or crushing from femurs and tibias. Bone marrow non-hematopoietic cells were isolated by flushing or crushing from femurs, tibias, and humeri followed by enzymatic digestion with collagenase type I (Gibco) and DNase I (Sigma).

Instrument

BD FACS Aria (BD Biosciences)

Software	FACS Diva 8.0.1 (BD Biosciences)
Cell population abundance	Abundance of relevant cell population within post-sort fraction and purity of sorted cell population was validated from reanalysis by flow cytometry.
Gating strategy	FSC-A/SSC-A was used for gating mononuclear cells. FSC-W/FSC-H was used for gating on singlets. Specific gating strategies are included in Supplementary Fig. 8.

☒ Tick this box to confirm that a figure exemplifying the gating strategy is provided in the Supplementary Information.
